# Does music counteract mental fatigue? A systematic review

**DOI:** 10.1371/journal.pone.0316252

**Published:** 2025-01-03

**Authors:** Cong Ding, Soh Kim Geok, He Sun, Samsilah Roslan, Shudian Cao, Yue Zhao

**Affiliations:** 1 Department of Sport Studies, Faculty of Education Studies, Universiti Putra Malaysia, Selangor, Malaysia; 2 Department of Sport Studies, Faculty of General Education Studies, Jiangsu Food and Pharmaceutical Science College, Huaian, Jiangsu, China; 3 School of Physical Education, Henan University, Kaifeng, China; University of Education, Lahore, PAKISTAN

## Abstract

**Introduction:**

Mental fatigue, a psychobiological state induced by prolonged and sustained cognitive tasks, impairs both cognitive and physical performance. Several studies have investigated strategies to counteract mental fatigue. However, potential health risks and contextual restrictions often limit these strategies, which hinder their practical application. Due to its noninvasive and portable nature, music has been proposed as a promising strategy to counteract mental fatigue. However, the effects of music on performance decrements vary with different music styles. Synthesizing studies that systematically report music style and its impact on counteracting performance decrements is crucial for theoretical and practical applications.

**Objectives:**

This review aims to provide a comprehensive systematic analysis of different music styles in counteracting mental fatigue and their effects on performance decrements induced by mental fatigue. Additionally, the mechanisms by which music counteracts mental fatigue will be discussed.

**Methods:**

A comprehensive search was conducted across five databases—Web of Science, PubMed, SCOPUS, SPORTDiscus via EBSCOhost, and the Psychological and Behavioral Sciences Collection via EBSCOhost—up to November 18, 2023. The selected studies focused solely on music interventions, with outcomes including subjective feelings of mental fatigue, physiological markers, and both cognitive and behavioral performance.

**Results:**

Nine studies met the predetermined criteria for inclusion in this review. The types of music interventions that counteract mental fatigue include relaxing, exciting, and personal preference music, all of which were associated with decreased subjective feelings of mental fatigue and changes in objective physiological markers. Cognitive performance, particularly in inhibition and working memory tasks impaired by mental fatigue, was countered by both relaxing and exciting music. Exciting music was found to decrease reaction time more effectively than relaxing music in working memory tasks. The physiological marker of steady-state visually evoked potential-based brain-computer interface (SSVEP-BCI) amplitude increased, confirming that exciting music counteracts mental fatigue more effectively than relaxing music. Behavioral performance in tasks such as arm-pointing, the Yo-Yo intermittent test, and the 5 km time-trial, which were impaired by mental fatigue, were counteracted by personal preference music.

**Conclusion:**

Relaxing music, exciting music, and personal preference music effectively counteract mental fatigue by reducing feelings of fatigue and mitigating performance decrements. Individuals engaged in mentally demanding tasks can effectively counteract concurrent or subsequent cognitive performance decrements by simultaneously listening to relaxing or exciting music without lyrics or by using music during recovery from mental fatigue. Exciting music is more effective than relaxing music in counteracting mental fatigue. Personal preference music is effective in counteracting behavioral performance decrements in motor control and endurance tasks. Mentally fatigued individuals could apply personal preference music to counteract subsequent motor control performance decrements or simultaneously listen to it to counteract endurance performance decrements. Future studies should specify and examine the effects of different music genres, tempos, and intensities in counteracting mental fatigue. Additionally, the role of music in counteracting mental fatigue in contexts such as work productivity, traffic accident risk, and sports requires further investigation, along with the underlying mechanisms.

## 1 Introduction

Mental fatigue is a psychobiological state induced by prolonged and sustained cognitive tasks [1, 2]. Individuals experiencing mental fatigue often report subjective feelings of "tiredness" and "unwillingness to continue the task at hand" [1–6]. This state is typically associated with higher perceived exertion and lower motivation, which correspondingly leads to detrimental effects on cognitive and physical performance [2, 7]. Mental fatigue significantly impacts daily life, with studies suggesting it is related to an increased risk of car accidents [8], decreased productivity [9], and impaired sports performance [1, 10]. Mental fatigue presents a considerable challenge in the sports domain [11]. Sun et al. systematically reviewed the impact of mental fatigue on athletes’ skill performance and found that mental fatigue is impaired by technical performance and decision-making skills—crucial components for achieving success in competitive environments [12]. Thus, exploring potential strategies that are free from health risks and applicable across various contexts is critical in daily work and sports competition.

Recently, Proost et al. systematically reviewed strategies for counteracting mental fatigue, categorizing them into behavioral strategies (e.g., physical activity, steam baths, or exposure to specific odors), physiological strategies (e.g., caffeine and creatine intake), and psychological strategies (e.g., increasing motivation) [13]. Although some of these strategies effectively counteract mental fatigue [13], physiological interventions, such as excessive caffeine consumption, have been associated with cardiovascular complications and disruptions in calcium balance, particularly for vulnerable populations like children and women of reproductive age [14]. Similarly, improper dosing of creatine supplements can lead to health risks such as muscle cramping and kidney stress [15]. These potential health risks and contextual constraints hinder the implementation of such counteracting strategies [16]. Additionally, while monetary incentives have been shown to counteract mental fatigue by increasing motivation [17], this external motivation may diminish individuals’ sense of competence and autonomy, ultimately reducing intrinsic motivation [18, 19].

Due to its noninvasive nature and applicability across multiple contexts, music has recently been suggested as a promising strategy for counteracting mental fatigue [4, 13, 20]. Music offers benefits beyond mere entertainment, as studies have reported that listening to music involves psychological processes that enhance attentional focus [21] and regulate emotions [22]. Furthermore, modern medicine has applied music therapy to treat children with psychiatric disorders [23]. Neuroscientific studies indicate that listening to music during exercise activates the brain’s reward centers and decreases activity in regions associated with fatigue [24]. These findings suggest that music not only influences cognitive and emotional regulation but also benefits individuals’ daily lives and well-being [25, 26].

The psychobiological model of endurance performance, proposed by Marcora et al., suggests that mental fatigue activates the anterior cingulate cortex (ACC) of the brain [2]. This activation increases adenosine transmission and correspondingly decreases dopamine transmission, leading to higher perceived exertion and reduced motivation, which in turn results in subsequent performance decrements [2, 27]. Notably, the ACC is also involved in attention regulation [28]. Mental fatigue impairs attention control [29–31], and since both mental fatigue and attention control processes overlap in the ACC, attention is proposed as another factor mediating the relationship between mental fatigue and performance [5].

Listening to music stimulates dopamine transmission [24], and since dopamine and adenosine transmissions interact [27], listening to music may reduce perceived exertion [32] and compensate for decreased motivation [33, 34], potentially counteracting mental fatigue. Additionally, music influences the perception of pain in the ACC [35]. Given that the ACC is involved in attention regulation [28], music’s role in regulating attention control may offer another pathway through which it counteracts performance decrements induced by mental fatigue.

The body of literature investigating music’s role in counteracting mental fatigue is growing [4, 13]. However, the effectiveness of different music styles in counteracting mental fatigue varies, ranging from relaxing music [20] to exciting music [36, 37], and personal preference music [38]. Moreover, the impact of music on counteracting mental fatigue-induced performance decrements extends beyond cognitive performance [20, 39] to include behavioral performance [38, 40], particularly in the sports domain [40, 41]. The practical implementation of music as a strategy to counteract mental fatigue has been limited due to the lack of systematic synthesis studies that provide a clear overview of music styles and their effects on diverse performance decrements.

Therefore, this systematic review aims to address this gap by identifying critical considerations related to different music styles that counteract mental fatigue and examining their effects on mitigating performance decrements in both cognitive and behavioral domains. The potential mechanisms by which music counteracts mental fatigue will also be discussed. This review also seeks to summarize the limitations of previous studies included in this review and offer suggestions for future research. Finally, the review provides practical implications for the implementation of music as a strategy to counteract mental fatigue-induced performance decrements in various daily life scenarios.

## 2 Methodology

This systematic review was conducted in accordance with the guidelines of the updated Preferred Reporting Items for Systematic Reviews and Meta-Analyses (PRISMA) statement [42, 43], and the protocol was registered with the International Platform of Registered Systematic Review and Meta-Analysis Protocols (INPLASY) under the registration number ID: 202250150.

### 2.1 Eligibility criteria

The eligibility criteria for this review encompassed studies published in English, with no restrictions on publication year. The inclusion criteria were based on the Population, Intervention, Comparison, Outcome, and Study Design (PICOS) framework [44]. Specifically, the inclusion criteria for this systematic review are outlined in Table 1. 1) For the population, the review focused on healthy individuals, without any restrictions on gender or age. 2) Regarding the intervention, only studies where participants were exposed solely to music were included; any combination of music with other interventions (e.g., massage) was excluded as it falls outside the scope of this review. 3) For the comparison, studies involving either no music or different styles of music listened to by participants were included. 4) Concerning outcomes, the review was restricted to studies assessing cognitive performance and behavioral performance affected by mental fatigue, where at least one aspect of mental fatigue evaluation (e.g., subjective feelings, cognitive/behavioral performance, physiological markers) was included. 5) Finally, in terms of study design, randomized controlled trials (RCTs), non-randomized controlled trials (nRCTs), and non-randomized non-controlled trials (nRnCTs) were included in this review.

**Table 1:** Inclusion criteria following PICOS conditions.

Category	Inclusion criteria
Population	Healthy individuals
Intervention	Participants listening to music
Comparison	No music, participants listening to different music style
Outcome	Mental fatigue level (subjective feelings, cognitive/behavioral performance, physiological markers)
Study design	RCT, nRCT, nRnCT

RCT randomized controlled trail, non-RCTs non-randomized controlled trail, nRnCT non-randomized non-control trail

### 2.2 literature search and selection

A systematic search for music and mental fatigue studies was conducted on November 18, 2023. The search was carried out across five databases: Web of Science, PubMed, SCOPUS, SPORTDiscus (via EBSCOhost), and the Psychological and Behavioral Sciences Collection (via EBSCOhost). Google Scholar and Medical Subject Heading (MeSH) terms in PubMed were employed to determine the appropriate search keywords. The search strategy involved using the Boolean operators "AND" and "OR" throughout the search process across the five databases. An overview of the keywords used in the different databases is presented in Table 2. Additionally, manual searches of the reference lists of the included studies and Google Scholar were conducted to ensure that no relevant studies were overlooked.

**Table 2:** Number of hits for the complete search strategy for the different databases.

Database	Complete search string	Hits (18/11/2023)
Web of Science	(ALL = (("Music" OR "Rap Music" OR "Music, Rap" OR "Hip Hop Music" OR "Hop Music, Hip" OR "Music, Hip Hop" OR "Jazz Music" OR "Music, Jazz" OR "Classical Music" OR "Music, Classical" OR "Songs" OR "Song" OR "Vocal Melody" OR "Melodies, Vocal" OR "Melody, Vocal" OR "Vocal Melodies" OR "Rock and Roll Music"))) AND ALL = (("mental fatigue" OR "mental exertion" OR "cognitive fatigue" OR "cognitive exertion" OR "mental exhaustion" OR "mental tiredness"))	52
PubMed	(("Music" OR "Rap Music" OR "Music, Rap" OR "Hip Hop Music" OR "Hop Music, Hip" OR "Music, Hip Hop" OR "Jazz Music" OR "Music, Jazz" OR "Classical Music" OR "Music, Classical" OR "Songs" OR "Song" OR "Vocal Melody" OR "Melodies, Vocal" OR "Melody, Vocal" OR "Vocal Melodies" OR "Rock and Roll Music")) AND (("mental fatigue" OR "mental exertion" OR "cognitive fatigue" OR "cognitive exertion" OR "mental exhaustion" OR "mental tiredness"))	41
SCOPUS	TITLE-ABS-KEY (("Music" OR "Rap Music" OR "Music, Rap" OR "Hip Hop Music" OR "Hop Music, Hip" OR "Music, Hip Hop" OR "Jazz Music" OR "Music, Jazz" OR "Classical Music" OR "Music, Classical" OR "Songs" OR "Song" OR "Vocal Melody" OR "Melodies, Vocal" OR "Melody, Vocal" OR "Vocal Melodies" OR "Rock and Roll Music") AND ("mental fatigue" OR "mental exertion" OR "cognitive fatigue" OR "cognitive exertion" OR "mental exhaustion" OR "mental tiredness"))	52
SPORTSDiscus	(("Music" OR "Rap Music" OR "Music, Rap" OR "Hip Hop Music" OR "Hop Music, Hip" OR "Music, Hip Hop" OR "Jazz Music" OR "Music, Jazz" OR "Classical Music" OR "Music, Classical" OR "Songs" OR "Song" OR "Vocal Melody" OR "Melodies, Vocal" OR "Melody, Vocal" OR "Vocal Melodies" OR "Rock and Roll Music")) AND (("mental fatigue" OR "mental exertion" OR "cognitive fatigue" OR "cognitive exertion" OR "mental exhaustion" OR "mental tiredness"))	296
Psychological and Behavioral Sciences Collection	(("Music" OR "Rap Music" OR "Music, Rap" OR "Hip Hop Music" OR "Hop Music, Hip" OR "Music, Hip Hop" OR "Jazz Music" OR "Music, Jazz" OR "Classical Music" OR "Music, Classical" OR "Songs" OR "Song" OR "Vocal Melody" OR "Melodies, Vocal" OR "Melody, Vocal" OR "Vocal Melodies" OR "Rock and Roll Music")) AND (("mental fatigue" OR "mental exertion" OR "cognitive fatigue" OR "cognitive exertion" OR "mental exhaustion" OR "mental tiredness"))	286

**Fig 1:**
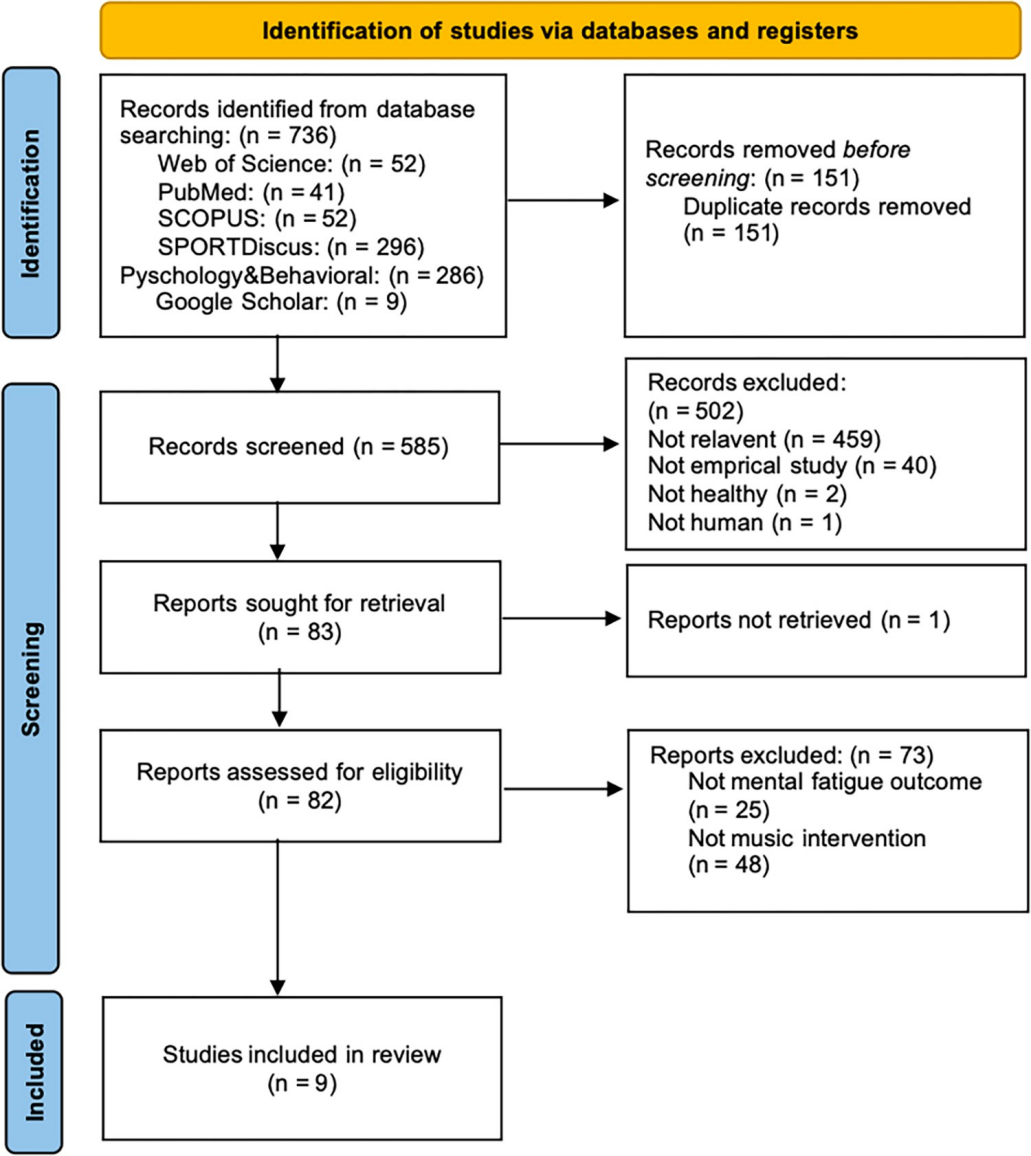
Flow diagram of the study selection process.

The study selection process is illustrated in Fig 1 and S1 File. After duplicates were removed, two independent evaluators (CD and HS) conducted a blinded screening of the studies based on the titles and abstracts. Full-text screening was then performed according to predetermined eligibility criteria. Any disagreements between the evaluators were resolved by consulting a third reviewer (KSG) to reach a final consensus.

### 2.3 Data extraction

Two independent reviewers (CD and HS) extracted data relevant to the aims of this review from the included studies on January 3, 2024, with accuracy validated by a third reviewer (KSG). The extracted information included:

1. The name of the first author and publication year.
2. Population characteristics: sample size, gender, age, population traits, and country.
3. Music intervention details: style of music, duration of the music intervention, whether music was used solely for recovery or simultaneously with task performance (single/multitasking), and overall duration.
4. Mental fatigue outcomes: performance domain, subjective evaluations, physiological markers, and cognitive/behavioral performance.

### 2.4 Risk of bias assessment

Two independent evaluators (DC and SH) assessed all studies included in this review. The Revised Cochrane Risk of Bias tool for randomized trials (RoB 2.0 for crossover trials) was used to evaluate the studies involving crossover trials [45]. Similarly, the revised Cochrane Risk of Bias tool for randomized trials (RoB 2.0) was applied for evaluating the RCTs [45]. The RoB 2.0 tool comprises five domains: "randomization process," "deviation from the intended interventions," "missing outcome data," "measurement of the outcome," and "selection of the reported result." One subdomain in RoB 2.0 for crossover trials specifically evaluates the "crossover effect" [45]. Two independent evaluators (DC and SH) adhered to the guidelines established by the Cochrane community and conducted the evaluations based on the specific signaling questions for each domain. After evaluating each study, an overall assessment of biases was generated for each included study. Any discrepancies were resolved through consultation with a third evaluator (KSG) until a consensus was reached.

## 3 Results

### 3.1 Study selection

A systematic literature search across five databases yielded 736 studies. After meticulous screening, 151 studies were excluded due to duplications. Following the review of titles and abstracts, 502 studies were deemed inconsistent with the objectives of this review and were excluded, leaving 83 studies. One study was removed because it could not be retrieved, resulting in 82 studies being subjected to full-text assessment. Of these, 73 studies were excluded based on predetermined inclusion criteria, and the final 9 studies were deemed eligible for inclusion in this systematic review. The selection process is illustrated in Fig 1 and S1 File.

### 3.2 Risk of bias

**Fig 2:**
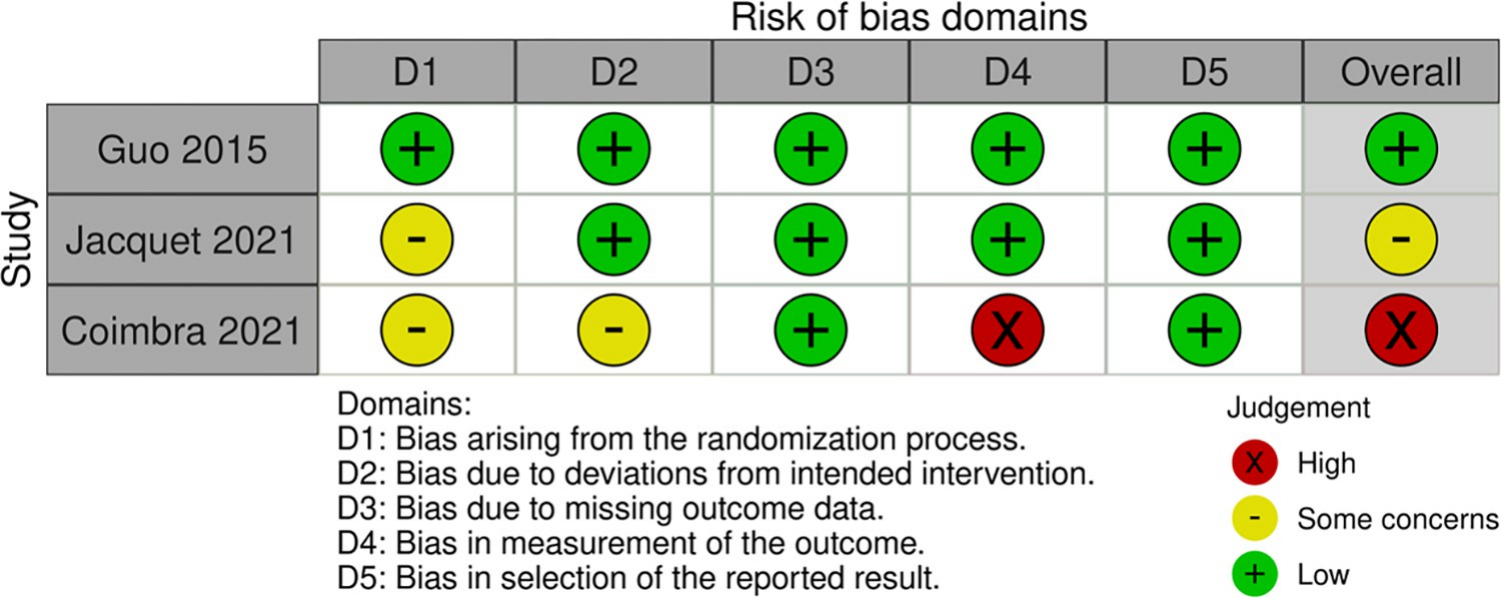
Risk of bias domains.

Nine studies included in this systematic review underwent risk of bias evaluation. Three studies utilizing a between-subjects study design (see Fig 2 and S2 File) were evaluated using the RoB 2.0 tool [20, 38, 46]. One study implemented a complete randomization procedure and was assessed as having a low risk of overall bias [20]. Another study did not report that the randomization process was evaluated, which led to some concern [38]. A third study was considered to have a high risk of bias because mental fatigue was not assessed immediately after the music intervention [46].

**Fig 3:**
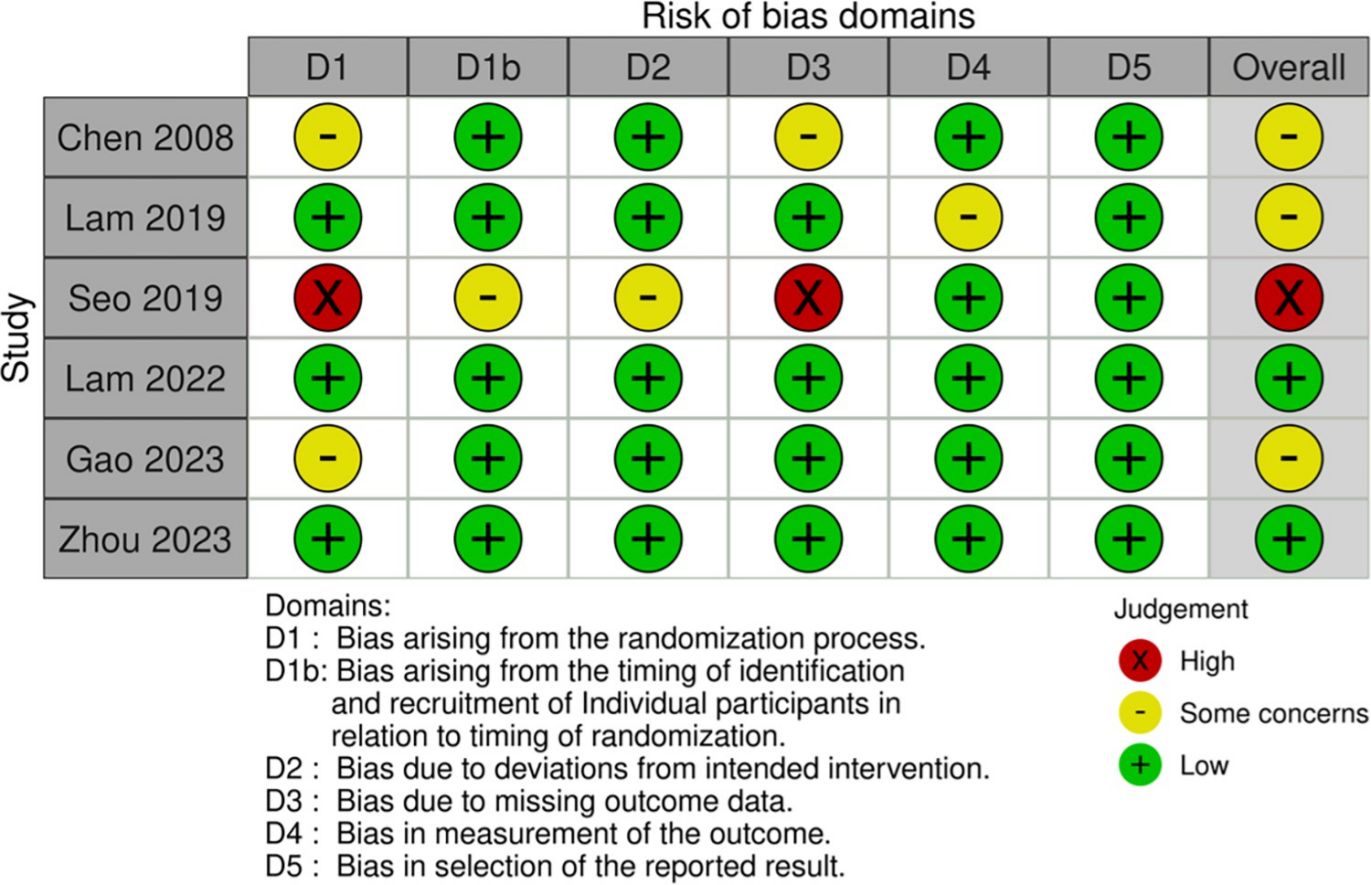
Risk of bias domains.

Six studies employing a within-subjects study design (see Fig 3 and S2 File) were evaluated using the RoB 2.0 tool for crossover trials [36, 37, 39–41, 47]. Two of these studies were evaluated as having a low risk of bias [37, 40]. Three studies [21, 32, 34] that did not implement a complete randomization procedure were assessed with some concern [36, 39, 41]. One study was evaluated as having a high risk of bias due to the investigator fully explaining the objective of the study to participants and incomplete reporting of mental fatigue outcome data [47].

### 3.3 Population characteristics

A total of 159 healthy participants were included across all studies. Five studies recruited both male and female participants [20, 36–38, 40]. Three studies focused exclusively on male participants [39–41]. One study focused solely on female participants [46]. Participant ages ranged from teenagers under 18 years old [46] to adults up to 35 years old [37]. Three studies focused on university students [20, 37, 39], while four studies recruited healthy adults [36, 38, 41, 47]. One study targeted recreational sports participants [40], and another study focused on volleyball players [46]. The participant countries varied, including China [20], Japan [39], and Brazil [46]. Detailed population characteristics are presented in Table 3.

**Table 3:** Overview of included publication details.

Study	Population characteristics	Intervention			Mental fatigue outcomes			
		Style	Period/ (multi)single tasking /duration		Performance domain	Performance outcome	Subjective evaluation	Physiological markers
Guo et al. (2015)	N: 36 university students; 18 ♂; 18 ♀; Age: 20.33 ± 1.26 yrs; Country: China	EG: Relaxing music CG: No music		P: T_0_ → T_2_; music & CT D: 60 mins	Cognitive: Inhibition response	EG vs CG Reaction time↓ Accuracy↔	EG vs CG VAS↓	EG vs CG: Go-P3 and NoGo-P3 amplitudes↑
Chen et al. (2008)	N: 8 ♂ university students Age: 22–25 yrs; Country: Japan	EG: Relaxing music CG: no music		P: T_1;_ music ◆ D: 15 mins	Cognitive: Working memory	EG vs CG Reaction time↓ Correct answers↑	EG vs CG VAS↓	EG vs CG Theta↓ Alpha↑ Beta↓
Zhou et al. (2023)	N: 8 university students 6 ♂; 2 ♀; Age: 18–35 yrs;	EG1: Exciting music EG2: Relaxing music CG: No music		P: T_1_ → T_2_; music & CT D: 15 mins	Cognitive: Working memory N-back tasks	EG vs CG Reaction time↓ EG1 vs EG2 Reaction time↓	EG vs CG VAS↓ EG1 vs EG2 VAS↓	NA
Jacquet et al. (2021)	N: 37 healthy adults 7 ♂; 30 ♀; Age: 20.6 ± 0.7 yrs;	EG: Preference music CG: Conversation		P: T_1;_ music ◆ D: 15 mins	Behavioral: Motor control Arm-pointing task	EG vs CG Duration↓ Errors↑	EG vs CG VAS↓	EG vs CG Theta↓ Alpha↑
Lam & Philips (2019)	N: 9 ♂ healthy adults Age: 22 ± 2.6 yrs;	EG: Preference music CG: no music		P: T_1_ → T_2_; music & ET	Behavioral: Endurance performance YYIRTL1	EG vs CG Distance↑	NA	NA
Lam et al. (2022)a	N: 9 ♂ recreational sports participants Age: 22 ± 2.6 yrs;	EG: Preference music CG: no music		P: T_1_ → T_2_; music & ET	Behavioral: Endurance performance YYIRTL1	EG vs CG Distance↑	EG vs CG BRUMS fatigue↓	NA
Lam et al. (2022)b	N: 9 recreational runners; 7 ♂; 2 ♀; Age: 21.1 ± 1.2yrs;	EG: Preference music CG: no music		P: T_1_ → T_2_; music & ET	Behavioral: Endurance performance 5 km time-trail performance	EG vs CG Completion time↓	EG vs CG BRUMS fatigue↓	Heart rate↑
Gao et al. (2023)	N: 6 healthy adults; 4 ♂; 2 ♀; Age: 20–25 yrs;	EG1: Exciting music EG2: Relaxing music CG: No music		P: T_0_ → T_2_; music & CT	NA	NA	NA	EG1&EG2 vs CG SSVEP amplitude↑ SNR↑ EG1 vs EG2 SSVEP amplitude↑ SNR↑
Coimbra et al. (2021)	N: 30 ♀ volleyball players; Age: < 18 yrs; Country: Brazilian	EG: Preference music CG: No music		P: after training; before bedtime;	NA	NA	EG vs CG VAS↔	NA
Seo et al. (2019)	N: 7 healthy adults; 5 ♂; 2 ♀; Age: 21–23 yrs;	21–23 yrs		P: T_0_ → T_2_; music & DT	During stimulate washing task	Comfort↑ Awareness↑ vigorous↑	EG vs CG Awareness: VAS↓	EEG signal: Alpha↑ Beta↓ Theta↑

N: number of participants; ♂: males; ♀: females; P: period; D: duration; T_0_:start point of the whole task; T_1_: point of mental fatigue manifested T_2_: end point of the task; →: from time point to time point; CT: cognitive task; ET: endurance task; DT: daily task &:listen to music simultaneous with the task; ◆: sole listening to music without any other task; EG, experimental group; CG, control group; MF, mental fatigue; ↓the value is significantly lower in experimental group/condition compare to the control group/condition; ↑the value is significantly higher in the experimental group/condition compared to control group/condition; ↔no significances between experimental and control group/condition; NA: not applicated; EEG: Electroencephalogram; YYIRTL1: Yo-Yo intermittent recovery test level 1; SDSD: standard deviation of adjacent successive heartbeat interphase difference value; TF: general frequency spectrum; LF: low-frequency power; HF: high-frequency power; HFnorm: corrected high-frequency power; SSVEP: the steady-state visually evoked potential; SNR: sign-to-noise

### 3.4 Styles of music intervention

A comprehensive search yielded nine studies examining the effect of music on counteracting mental fatigue. The music styles included relaxing, exciting, and personally preferred music [20, 36–41, 46, 47]. Generally, relaxing music features a slow tempo, less than 90 beats per minute (BPM), and induces a soothing and relaxing effect [37, 48]. Four studies investigated the effect of relaxing music compared to no music on cognitive performance, including inhibition response, reasoning, and working memory [20, 36, 37, 39]. Recently, Zhou et al. and Gao et al. explored the effects of different music styles on counteracting mental fatigue [36, 37]. Zhou et al. compared relaxing and exciting music in relation to cognitive performance in working memory [37], while Gao et al. compared relaxing and exciting music and used physiological markers of SSVEP-BCIs to examine the effects of music on counteracting mental fatigue [36]. Exciting music, characterized by a fast tempo generally above 120 BPM [37, 48], was found to reduce reaction time on cognitive tasks and increase SSVEP amplitude signals compared to relaxing music [37].

Personal preference music was defined based on participants’ choices to enhance their pleasure. Three studies [10, 20, 33, 34, 36] investigated the effect of personally preferred music on mental fatigue-induced motor control and endurance performance decrements [38, 40, 41]. Lam et al. instructed participants to listen to their preferred music while performing the Yo-Yo interval intermittent task under conditions of mental fatigue [41]. Subsequently, Lam et al. asked mentally fatigued participants to listen to their preferred music during the Yo-Yo interval intermittent test and a 5 km time trial endurance task [40].

### 3.5 Subjective and physiological markers of mental fatigue

Nine studies examined the effect of music on counteracting mental fatigue through subjective evaluation, performance changes, and physiological markers [20, 36–41, 46, 47]. The visual analog scale (VAS), known for its reliability and ease of interpretation, was widely used for subjective evaluations in these studies [49]. Guo et al., Chen et al., and Zhou et al. reported that participants exposed to music conditions experienced less mental fatigue compared to those without music [20, 37, 39]. Additionally, Zhou et al. found that listening to exciting music reduced mental fatigue more effectively than relaxing music [37]. The Brunel Mood Scale (BRUMS) fatigue subscale is another measure of subjective mental fatigue. Zhou et al. and Lam et al. reported that participants experienced significantly lower levels of mental fatigue on the BRUMS fatigue subscale when exposed to music conditions compared to no music conditions [37, 41].

Physiological markers offer objective insights into the effects of music on mental fatigue. Electroencephalography (EEG) is a real-time and sensitive method for measuring mental fatigue [50]. Chen et al. and Jacquet et al. found that participants exposed to music conditions showed a decrease in theta waves and an increase in alpha waves [38, 39], indicating that music effectively counteracts mental fatigue from a physiological perspective. Additionally, event-related potentials (ERPs) revealed that participants under music conditions exhibited an increased P3 amplitude in the Go/NoGo task [20]. Steady-state visually evoked potential-based brain-computer interface (SSVEP-BCI) is a more direct and sensitive method for detecting brain activity [51]. Gao et al. reported that participants in music conditions had significantly higher SSVEP amplitudes compared to those without music [36]. Consistent with Zhou et al.’s findings, which indicated that exciting music was more effective at counteracting mental fatigue than relaxing music [37], the SSVEP amplitudes were significantly higher under the exciting music condition compared to the relaxing music condition [36].

### 3.6 Music counteracts performance decrement

Besides affecting mental fatigue through subjective feelings and physiological markers, music also counteracts mental fatigue by mitigating performance decrement, which has more direct implications for individuals’ daily lives.

#### Cognitive performance

Three studies examined cognitive performance using the Go/NoGo task, the arithmetic two-digit addition task, and the N-back task [20, 36, 37, 39]. All three cognitive tasks are sensitive to cognitive performance and require participants to respond as quickly and accurately as possible [52]. The Go/NoGo task primarily assesses inhibition and response control, requiring rapid action to Go stimuli while suppressing the prepotent urge to respond to NoGo stimuli [53]. Successful performance relies on effective top-down control from the prefrontal cortex [54], which regulates impulsive responses and ensures selective attention to relevant stimuli [55]. Following mental fatigue induction, participants’ reaction times significantly increased in the control group (from 500 ms to 520 ms) but remained unchanged in the music group (from 502 ms to 498 ms) [20].

The N-back task involves working memory, updating, and attention in cognitive processing [56, 57]. Participants must maintain a representation of the target stimulus N items back in memory, compare it to the current stimulus, and update their representation while retaining relevant information from previous presentations [56]. After 60 minutes of N-back mental fatigue induction, participants’ response times in the music condition differed significantly (F(2, 20) = 55.21, P < 0.05) compared to those in the no-music condition [37]. Moreover, the response time in the N-back task was significantly shorter in the exciting music group compared to the relaxing music group [37].

The arithmetic two-digit addition task is a relatively straightforward task that engages multiple cognitive processes, including working memory, attention, and processing speed [58]. Participants needed to remember the first number while processing the second, perform the addition calculation, and retrieve the answer from memory within a limited time frame. After 15 minutes of listening to relaxing music, mentally fatigued participants’ reaction times to arithmetic addition were significantly shorter, and the number of correct answers improved compared to the no-music condition [39].

#### Behavioral performance

Three studies investigated behavioral performance using the arm-pointing test, the Yo-Yo intermittent recovery test, and a 5 km treadmill time trial [38, 40, 41]. Motor control tasks require participants to integrate visual and proprioceptive information into the central nervous system and maintain accurate pointing despite external stimuli [59, 60]. This task necessitates efficient communication between various brain regions involved in sensory processing, movement planning, and motor execution [60]. Mentally fatigued participants who listened to music for 15 minutes performed the motor control task for a shorter duration compared to those in the control group [38].

The Yo-Yo intermittent recovery test assesses endurance fitness, energy recovery capacity, and strategic pacing. Participants need to push themselves during the intervals, manage their exertion, and optimize their recovery during the rest periods [61]. This condition requires cognitive control, attention to internal bodily cues, and decision-making skills [62]. Mentally fatigued participants who listened to their preferred music covered longer distances (564 ± 127 m) compared to those who did not listen to music (496 ± 112 m) [41]. Similarly, participants in the music condition covered longer distances compared to those in the no-music condition (p = 0.007) [40].

The 5 km treadmill trial is an endurance performance task involving multiple cognitive processes, such as motivation, attention, focus, pacing strategy, and pain tolerance [63]. Athletes must maintain high levels of effort and focus throughout the run, manage their pacing to avoid exceeding their capacity, and push through fatigue and discomfort [63]. Mentally fatigued participants completed the time trial in a shorter time (23.1 ± 2.4 min) compared to those without music (24.1 ± 3.2 min) [40].

## 4 Discussion

This systematic review aimed to outline the current knowledge of how music counteracts mental fatigue, focusing on subjective evaluation, physiological markers, and cognitive/behavioral performance. It also examined the impact of different music styles on mitigating mental fatigue-induced performance decrements in both cognitive and behavioral domains. Additionally, the review discusses the potential mechanisms by which music counteracts mental fatigue.

### 4.1 Style of music intervention

Interestingly, although the styles of music are diverse in modern society, all the studies included in this review that investigated music’s effect on counteracting mental fatigue focused on relaxing music, exciting music, and personal preference music. The first investigation that applied relaxing music to counteract mental fatigue was conducted by Chen et al., who used arithmetic addition to simulate daily work and employed relaxing music after a prolonged cognitive task to create a work-rest schedule [39]. Their study suggested that relaxing music induces more relaxation than a no-music condition. This finding aligns with Labbé et al., who reported that relaxing music reduced anxiety and anger while increasing relaxation compared to silence [64].

Additionally, Khalfa et al. reported that relaxing music reduced salivary cortisol levels after a psychologically stressful task [65]. Since salivary cortisol levels are associated with mental fatigue [66], this reduction provides physiological evidence that relaxing music effectively counteracts mental fatigue. Similarly, Chen et al. and Guo et al. investigated the effects of relaxing music on mental fatigue. Participants performed 60 AX-continuous performance tasks with relaxing music, significantly increasing Go-P3 and NoGo-P3 amplitudes [20]. The P3 amplitudes are involved in the allocation of attentional resources [67]. This finding supports Sun et al.’s proposed improved psychobiological model of endurance performance, which suggests that attention mediates the relationship between mental fatigue and performance [5]. Zhou et al. compared relaxing and exciting music in relation to cognitive tasks using the N-back test. They found that participants listening to exciting music experienced greater counteraction of mental fatigue than those listening to relaxing music, with these effects also manifested in shorter reaction times [37].

Consistent findings were reported by Gao et al., who found that participants listening to exciting music had increased SSVEP-BCI amplitudes compared to those listening to relaxing music [36]. SSVEP amplitudes can track the attentional field by measuring changes in response to specific flickering stimuli [51]. These findings suggest that exciting music may regulate attention-related cognitive resources more effectively than relaxing music. This supports Sun et al.’s improved model, which implicates attention as a factor mediating mental fatigue and performance [5]. The greater effectiveness of exciting music in counteracting mental fatigue compared to relaxing music might be attributed to its high-tempo nature, which stimulates more dopamine transmission [24], thereby enhancing attention regulation. Considering that music affects the anterior cingulate cortex (ACC) and mental fatigue activates the ACC [2, 27, 35], it is possible that music influences mental fatigue through pathways involving the ACC. However, further studies are needed to investigate the effects of exciting versus relaxing music on dopamine transmission and their interactive mechanisms.

Notably, all studies that investigated the effects of relaxing and exciting music on mental fatigue during cognitive tasks used instrumental music without lyrics [20, 36, 39]. Previous studies have reported that music with lyrics can lead to poorer performance on cognitive tasks because the lyrics divert limited attentional resources [68]. Future research is needed to determine whether music with lyrics has less or no effect on mental fatigue-induced cognitive performance decrements.

All included studies that examined the effects of music on behavioral performance used personally preferred music [10, 20, 34, 38, 40, 41]. This is consistent with sports literature suggesting that participants perform better when listening to personally preferred music compared to non-preferred music [69, 70]. As individuals have varied music preferences, listening to personally preferred music may induce more pleasure. According to the psychological model of endurance performance, dopamine and adenosine interactions play a role [2, 27]. Music selected based on personal preference might enhance pleasure and stimulate dopamine transmission more than non-preferred music. However, the specific pathways through which personal preference music counteracts mental fatigue, including interactions with dopamine or adenosine in the ACC, remain unclear. Further exploration in this area could provide valuable insights into the mechanisms by which music counteracts mental fatigue.

### 4.2 Subjective evaluation and physiological markers

The subjective evaluation of mental fatigue is widely recognized as a reliable and feasible approach in various fields, including neuroscience and psychology [71]. Five of the included studies [20, 22, 32–36] reported that listening to music significantly decreased the subjective feeling of fatigue compared to no music [20, 37, 39–41]. In contrast, Coimbra et al. found no effect of preferred music on subjective recovery in teenage female volleyball players after training and before bedtime [46]. Their evaluation of subjective recovery before training was methodologically less controlled and assessed as high risk of bias. Further studies are needed to confirm the effectiveness of music in counteracting mental fatigue among volleyball players.

Seo et al. instructed healthy adults to perform a daily task, simulating dishwashing, and reported a reduction in mental fatigue with music compared to no music [47]. Although this study was evaluated as high risk of bias, its findings align with the notion that music effectively counteracts mental fatigue. Nonetheless, further research is needed to confirm these results under similar conditions.

Physiological markers offer an objective and direct method for measuring mental fatigue. Changes in theta activity in the frontal, central, and posterior regions are considered robust biomarkers of mental fatigue [72], while changes in alpha wave activity are regarded as secondary biomarkers [72]. Lower theta levels are generally associated with higher levels of alertness and focus, whereas increased alpha activity reflects reduced vigilance and sensory processing, indicating greater relaxation [73]. Both studies reported that participants who listened to music exhibited decreased theta waves and increased alpha waves compared to those in the no-music condition [38, 39].

### 4.3 Music counteracts performance decrement

#### Cognitive performance

Studies evaluated cognitive performance using the Go/NoGo task, N-Back, and arithmetic addition, focusing on inhibition response, working memory, and reasoning [20, 37, 39]. Despite the variations in tasks, each involves attentional processes. Prolonged cognitive tasks deplete mental resources, leading to insufficient attention to focus on the task and, consequently, a decrease in cognitive performance [74]. Kok et al. suggested that attentional resources are the primary determinant of cognitive task performance, and a lack of availability directly impacts performance decrements [75, 76].

The reduction in attentional resources leads to deficits in attention control and, thus, a decrease in cognitive performance. Music can regulate attentional resources more effectively [67]. Gao et al. applied event-related potentials (ERPs) during the Go/NoGo task and found that mental fatigue increased Go-P3 and NoGo-P3 amplitudes, where P3 is related to attentional resource allocation [36]. Go-P3 reflects the allocation of attentional resources to stimuli, while NoGo-P3 represents the inhibition of responses [36]. Thus, listening to music may help participants allocate resources more effectively toward attention, explaining how music counteracts mental fatigue and performance decrements. Consistent findings were reported by Chen et al. [42], who instructed participants to listen to music and found improved reaction times and accuracy in arithmetic performance after music compared to no music [39]. Participants also reported exerting less effort, maintaining better attention, and being more alert [39].

Physiologically, mental fatigue activates the anterior cingulate cortex (ACC), a crucial brain region involved in reward evaluation and attention regulation [77]. A prevailing theory suggests that mental fatigue disrupts dopaminergic networks within the ACC. Prolonged neural activity associated with fatiguing tasks increases adenosine, a byproduct of ATP breakdown [27, 35, 77]. Adenosine and dopamine receptors are located within the ACC [78]. This spatial overlap suggests that adenosine might interfere with dopaminergic signals, contributing to reduced ACC activity during mental fatigue. This, in turn, could lead to increased adenosine transmission, higher fatigue perception, and hindered resource allocation. Music regulates attentional resources [79], potentially alleviating mental fatigue-induced performance decrements. However, further research is needed to better understand the mechanisms through which dopamine transmission counteracts mental fatigue.

Zhou et al. investigated the effect of different music tempos on cognitive performance impairment caused by mental fatigue [37]. They reported that participants in the exciting music condition demonstrated faster reaction times than those in the relaxing music condition [37]. This finding suggests that exciting music, due to its higher tempo, might activate the ACC more strongly, leading to enhanced dopamine transmission and better resource allocation to attention. However, whether exciting music induces more dopamine transmission than relaxing music remains to be explored, and further research is required to investigate the specific mechanisms underlying this phenomenon.

#### Behavioral performance

Studies have investigated the effect of music on counteracting mental fatigue in terms of behavioral performance, specifically motor control and endurance [38, 40, 41]. Jacquet et al. assessed the effects of mental fatigue on motor control performance using an arm-pointing task [38]. Motor control, initiated by the central nervous system and involving muscle recruitment along the skeletal system, requires precise movement and engages cognitive attention, planning, and motivation [60]. Jacquet et al. suggested that listening to personal preference music compensates for the dopamine system’s response to mental fatigue, allowing participants to regulate their attention resources more effectively and perform the arm-pointing task for a shorter duration [38]. This finding aligns with the improved psychobiological model of endurance performance proposed by Sun et al., which suggests that attention mediates the relationship between mental fatigue and performance [5].

Two additional studies focused on behavioral performance in endurance tasks, specifically the Yo-Yo interval intermittent test and the 5-km time trial [40, 41]. According to the psychobiological model of endurance performance, mental fatigue activates the anterior cingulate cortex (ACC), leading to increased adenosine levels and decreased dopamine transmission [2, 5, 27]. Elevated adenosine increases perceived exertion, while reduced dopamine leads to decreased motivation and attention [2, 5, 27]. During endurance exercise, participants’ attention is directed either towards bodily signals (association) or away from these signals to focus on the environment and external cues (dissociation) [80]. The perceived exertion arising from bodily signals may be mitigated by music, which could shift attention from bodily sensations to the music, thus promoting dissociation. This dissociation might be a pathway through which music counteracts mental fatigue during endurance performance. Additionally, music may affect the ACC, reducing the perception of effort, thereby decreasing perceived exertion and improving task-focused attention, which counters endurance performance decrement [35]. Although the interaction between adenosine and dopamine is recognized, the complete mechanism by which music counteracts mental fatigue-induced performance decrement remains under exploration. Further research in this area could enhance understanding of how music mitigates mental fatigue.

### 4.4 Significance, limitations and future directions

This review provides insights into how different music styles affect mental fatigue-induced performance decrements. Listening to relaxing music, exciting music, and personal preference music effectively reduces the feeling of mental fatigue. Employing background music, such as relaxing or exciting music without lyrics, can help mitigate mental fatigue and counteract cognitive performance decrements for individuals engaged in mental tasks, such as students and workers. In sports settings, players experiencing mental fatigue may benefit from listening to music that is of personal preference to aid recovery and enhance both mental fatigue reduction and motor control performance. Additionally, mentally fatigued athletes undergoing endurance exercises can use personal preference music to counteract performance decrements.

Despite the insights provided by this review, certain limitations remain. Due to the heterogeneity of performance outcomes and the insufficient number of included studies, a meta-analysis of the effects of different music styles on performance decrements was not feasible. A meta-analysis could be conducted in the future if a sufficient number of studies become available.

Regarding music styles, while relaxing music, exciting music, and personal preference music are effective in counteracting mental fatigue and performance decrements, the diverse range of music styles in the real world remains underexplored. Terms like relaxing music and exciting music are general; specific music genres such as classical, jazz, rock, pop, hip-hop, rap, and electronic dance music, which are listed in the MeSH database of PubMed, have not been examined. As Koelsch et al. suggested, methodological recommendations for music research should include accurate characterization of musical and acoustical features, including genre, tempo, instrumentation, and loudness [81]. Providing these specific details could offer more direct practical implications for individuals aiming to counteract mental fatigue.

Additionally, while preliminary studies have investigated the effects of music on cognitive and behavioral performance decrements, further research is needed to explore the impact of music on productivity, long-duration driving risks, and sports performance, given the impairments associated with mental fatigue.

Lastly, investigating the underlying mechanisms by which music counteracts mental fatigue is crucial. Although Sun et al. proposed that attention mediates the relationship between mental fatigue and performance, the complete mechanism involving music’s effects on dopamine transmission, adenosine transmission, and their interaction with attention regulation in the ACC still requires robust evidence to be confirmed.

## 5 Conclusion

Relaxing, exciting, and personal preference music effectively counteract mental fatigue. For individuals performing cognitive tasks, listening simultaneously to relaxing and exciting music without lyrics reduces the feeling of mental fatigue and counters cognitive performance decrements induced by mental fatigue. Exciting music has been found to be more effective than relaxing music in mitigating mental fatigue and enhancing cognitive performance. When mental fatigue manifests, listening to either relaxing or exciting music can aid in reducing fatigue and counteracting performance decrement on subsequent cognitive tasks.

In terms of behavioral performance, individuals experiencing mental fatigue can benefit from listening to music that is of personal preference to alleviate mental fatigue and enhance motor control performance. Similarly, mentally fatigued athletes engaged in endurance exercises can use personal preference music to reduce fatigue and counteract performance decrements. The included studies support the improved psychobiological model of endurance performance, which suggests that attention is a key factor mediating mental fatigue and performance. However, further research is needed to understand the mechanisms by which music counteracts mental fatigue fully.

## Supporting information

S1 Checklist PRISMA 2020 checklist. (DOCX)

S1 File Studies identification. (XLSX)

S2 File Evaluation of included studies. (XLSX)
